# Clinical Utility of a Digital Therapeutic Intervention in Indian Patients With Type 2 Diabetes Mellitus: 12-Week Prospective Single-Arm Intervention Study

**DOI:** 10.2196/41401

**Published:** 2022-10-31

**Authors:** Rajeev Chawla, Shalini Jaggi, Amit Gupta, Ganapathi Bantwal, Suhas Patil

**Affiliations:** 1 North Delhi Diabetes Centre Delhi India; 2 Lifecare Diabetes Centre Delhi India; 3 Center for Diabetes Care Greater Noida India; 4 Terrals Technologies Private Ltd Bangaluru India

**Keywords:** HbA1c, type 2 diabetes, digital therapeutics, fasting blood glucose, postprandial blood glucose, mHealth, digital health intervention, glycemic control, mobile health

## Abstract

**Background:**

Patients with type 2 diabetes mellitus (T2DM) having elevated levels of blood glucose and glycated hemoglobin (HbA_1c_) are at higher risk of macro- and microvascular complications. Nonetheless, the goal of achieving glycemic control cannot be met with the use of pharmacotherapy alone. The recent emergence of digital therapeutic tools has shown the possibility of improving the modifiable risk factors and self-management of diabetes.

**Objective:**

The aim of this study was to examine the clinical utility of a digital therapeutic intervention as an add-on therapy to achieve glycemic control in patients with T2DM.

**Methods:**

This was a 12-week prospective, single-arm digital intervention study in patients with T2DM receiving regular antidiabetic treatment. The eligibility criteria included male and female patients with HbA_1c_≥6.5%, functional English literacy, and a mobile phone capable of running the intervention app. Outcome measures of the study were mean changes in HbA_1c_, fasting blood glucose (FBG), postprandial blood glucose (PPBG), BMI, and Homeostatic Model Assessment for Insulin Resistance (HOMA-IR) index at the end of 12 weeks.

**Results:**

A total of 128 participants completed the study period of 12 weeks. There were 54.7% (70/128) men and 45.3% (58/128) women with a mean age of 48.48 years (SD 10.27). At the end of 12 weeks, the mean change in HbA_1c_, FBG, PPBG, and BMI for the overall study population was –0.84% (*P*<.001), –8.39 mg/dl (*P*=.02), –14.97 mg/dl (*P*<.001), and –0.24 kg/m^2^ (*P*=.06), respectively. Among the participants showing improvement in the HbA_1c_ value at the end of 12 weeks (responders), the mean change in HbA_1c_, FBG, PPBG, and BMI was –1.24% (*P*<.001), –12.42 mg/dl (*P*=.003), –21.45 mg/dl (*P*<.001), and –0.34 kg/m^2^ (*P*=.007), respectively. There was an increase in HOMA-IR values for the overall study population (0.54, *P*=.29). HbA_1c_ response showed a significant association with a baseline HbA_1c_ level ≥7.5%, no prior history of smoking, and no prior COVID-19 infection, as well as with higher levels of program engagement.

**Conclusions:**

A digital therapeutic intervention when used alongside standard medications significantly reduces HbA_1c_, FBG, and PPBG levels in patients with T2DM.

## Introduction

### Background

Type 2 diabetes mellitus (T2DM) is a metabolic endocrine disorder that is characterized by continually elevated blood glucose levels. In India, it is estimated that there are approximately 74.2 million people with diabetes, accounting for 13.8% of the global prevalence [1]. It is projected that by 2045, India will have 124.9 million adults with diabetes, corresponding to 16% of the global burden of the disease [1]. Even more alarming, the age of onset of diabetes in the Indian population is younger and with a considerably lower BMI as compared with those of other racial-ethnic groups [2,3]. This is typically classified as the “Asian Indian or South Asian phenotype,” represented by higher levels of belly fat, less muscle mass, and increased insulin resistance, even at low BMI [4].

As a chronic progressive disease, the medical management of T2DM often necessitates the intensification of medications during the course of treatment. However, there is also compelling evidence to support the role of therapeutic lifestyle changes such as weight loss, dietary restrictions, exercise, adequate sleep, and health coaching in the effective management of diabetes without requiring intensification of the medications [5-10]. The participation of patients by way of self-monitoring of blood glucose (SMBG), strict compliance to dietary restrictions, and exercise routine, along with adequate sleep to abate stress is essential in avoiding disease progression and the long-term complications associated with T2DM [11].

In recent years, digital technologies have attracted increasing attention to enable lifestyle modification by patients and help them achieve the goal of self-management of diabetes to reduce the disease burden [11-15]. As a mostly remote intervention form, digital therapeutic interventions (DTIs) may not be as impactful as in-person counseling and follow-ups, but have the advantage of ease of communication, anytime-anywhere accessibility, and availability of information. A DTI overcomes the barrier of physical transportation, particularly in restricted conditions such as during the COVID-19 pandemic [12-15]. The machine learning capabilities through cloud computing and interactive interfaces of smartphones have made behavior modification possible based on personalized nudges, information sharing, and communication [12-14]. The ease of intervention along with autonomy and constant reminders can keep a patient motivated in achieving the desired therapeutic goals. Several studies have demonstrated the role of digital technology such as digital therapeutic platforms, telehomecare systems, digitally enhanced diabetes self-management, education and support programs, as well as smartphone-based integrated online real-time diabetes care systems in the effective management of T2DM, without requiring escalation of existing medication [12-17]. However, there is also substantial variation in the reported intervention approaches and observed changes in outcome parameters. In view of these variations of the impact of DTIs, there is a requirement of measuring the effectiveness of a particular DTI approach before it can be scaled up for a larger target population.

### Objectives

The goal of our study was to assess the utility of a DTI in achieving glycemic control in Indian patients with T2DM. We hypothesized that lifestyle and behavior modification through the DTI would improve glycated hemoglobin (HbA_1c_) of the participants from the preintervention level. Additional parameters to assess the effectiveness of the intervention included changes in fasting blood glucose (FBG) and postprandial blood glucose (PPBG) levels, BMI, and the Homeostatic Model Assessment for Insulin Resistance (HOMA-IR) index. Moreover, we aimed to assess the potential relationships between user engagement and outcome results.

## Methods

### Study Design, Sample Size Calculation, and Eligibility Criteria

This was a 12-week, prospective, single-arm intervention study in Indian patients with T2DM receiving regular antidiabetic treatment from their physician. The sample size calculation was based on the study of Bollyky et al [18]. To detect a mean change in HbA_1c_ levels of –0.4% with a sample SD of 1.5, using a 5% level of significance, 80% power, and correlation coefficient of 0.5 with a two-tailed *t* test of paired mean difference, a sample size of 113 was needed. Assuming a dropout rate of 20%, a sample size of 136 was chosen for this study. The sample size was calculated using SAS software version 9.4 (SAS Institute Inc).

The eligibility criteria included male and female patients with T2DM, aged 18-65 years, HbA_1c_≥6.5%, functional English literacy for use of the mobile app, and a smartphone capable of running the intervention app. Participants were also required to be on a stable dose of antidiabetic medications at the time of entry in the study, with the expectation to remain on the same stable dose during the study period. Subjects were excluded if they were unable or unwilling to provide informed consent and comply with the protocol procedures; had a previous diagnosis of gestational diabetes, myocardial infarction, or stroke; were pregnant or lactating; and had restricted physical movements as per the clinical judgment of the treating physician. The study was conducted between October 2021 and March 2022 at three metropolitan outpatient clinics catering specifically to patients with diabetes.

### Ethics Considerations

The study was conducted according to the ethical principles stated in the latest version of the Helsinki Declaration and the applicable guidelines for good clinical practice. Ethics committee approval for this study was obtained from the Good Society for Ethical Research–Independent Ethics Committee for Biomedical Research, Delhi (approval numbers: GSER/2021/BMR-AP/035, GSER/2021/BMR-AP/037, and GSER/2021/BMR-AP/039 dated October 8, 2021). The outpatient subjects who were willing to participate in the study were asked to sign a written informed consent form to assess their eligibility. Those unwilling to participate continued to receive the usual standard of care at the clinics.

### Study Intervention

#### Digital Therapeutic App

The DTI model for this study was developed by Phable Care, India. The intervention approach connects patients with doctors through smartphone apps. The patient app can be connected with Bluetooth-enabled devices such as a glucometer or blood pressure monitor, as well as with other fitness applications such as Google Fit and Apple Health. Any vital measurement data from these devices are transmitted to the patient app. These data are shared with the connected physician for any real-time intervention necessary through the doctor app. Depending on the deviation of measured vitals from normal values, both the patients and doctors are informed via the study app. However, medication reminders for the patients are created from the doctor’s prescription and any changes therein. Further, based on the user’s disease profile and vitals-related data, personalized health education is provided. The health education–related content and communication are provided based on various behavior change theories (eg, the transtheoretical model of behavior change, nudge theory), which were integrated in the form of nudges, incentives, and reminders. The DTI model can be further intensified by a care team of nutritionists, physical trainers, and health coaches. For this study, we used the smartphone app plus care team approach.

#### Care Team Intervention

Participants who met the eligibility criteria and provided informed consent were included in the study. The participants were asked to download the app from either the Google Play or Apple App store as per the compatibility of their mobile phones. Participants were also provided with the Roche Accu-Chek Instant Glucometer, test strips, and lancets for SMBG. Thereafter, they were trained on the use of the study app and glucometer. During the study period of 12 weeks, participants received notifications and reminders via the study app to complete various study-specific tasks, including SMBG at fasting and 2 hours postmeal at least once a week, as well as participating in digital consultations with a doctor (weeks 1, 5, 9), dietician (weeks 1, 3, 5, 7, 9, 11), and exercise coach (weeks 1, 5, 9) to receive personalized instructions for the management of their disease condition. Beyond this structured intervention, interested participants could speak with care team members via phone call anytime as per need. Participants used the app to register their daily health activities personalized by their care provider as well as for the logging of weekly SMBG readings. Furthermore, they received patient education material on a weekly basis to strengthen their knowledge and awareness about the self-management of diabetes. At the end of the study period, participants were required to undergo laboratory testing and complete a study-specific questionnaire to evaluate the impact of the study intervention on the self-management of T2DM.

### Outcome Measures

The outcome measures of the study were mean changes in HbA_1c_, FBG, PPBG, BMI, and HOMA-IR index at the end of the 12-week intervention from the respective baseline values. The data pertaining to the above variables were collected from the laboratory reports and SMBG readings. The outcomes were measured based on a pre-post change in the values of these variables. Participants were classified as “responders” if they showed an improvement in the HbA_1c_ value at the end of 12 weeks and as “nonresponders” if the HbA_1c_ value had either remained unchanged or had increased at the end of 12 weeks.

Based on the level of participation, participants were also divided into three groups: low-engagement group (50%-60% engagement), medium-engagement group (61%-80% engagement), and high-engagement group (>80% engagement). Program engagement was assessed subjectively by the health coaches based on the participant’s response to communication, dietary changes, physical activities, and SMBG. Participation was measured quantitatively in terms of the frequency of engagement components as reported by the participants themselves. A monthly report of progress and participation was shared with participants based on these data. The engagement score for final analysis was calculated from the arithmetic sum of individual scores and then converted into percentages. A questionnaire was further administered to the participants at the end of the intervention to assess the impact of the program on diabetes self-management. The responses were summarized in simple arithmetic measures.

### Statistical Analysis

Continuous variables are summarized by the arithmetic mean (SD). A paired-sample *t*-test or repeated-measures ANOVA (as applicable) was used to compare the change in the mean values of parameters at the end of the study. Categorical variables are summarized using frequencies and percentages. Statistical analysis was performed using the Pearson *χ*^2^ or Fisher exact test, as appropriate, to test the association between the variables. Two-sided *P*<.05 was considered statistically significant. Simple logistic regression was performed to evaluate the association of each baseline characteristic with the achievement of HbA_1c_ response. Statistical analysis was performed using SAS version 9.4 (SAS Institute Inc).

## Results

### Baseline Characteristics

**Figure 1:**
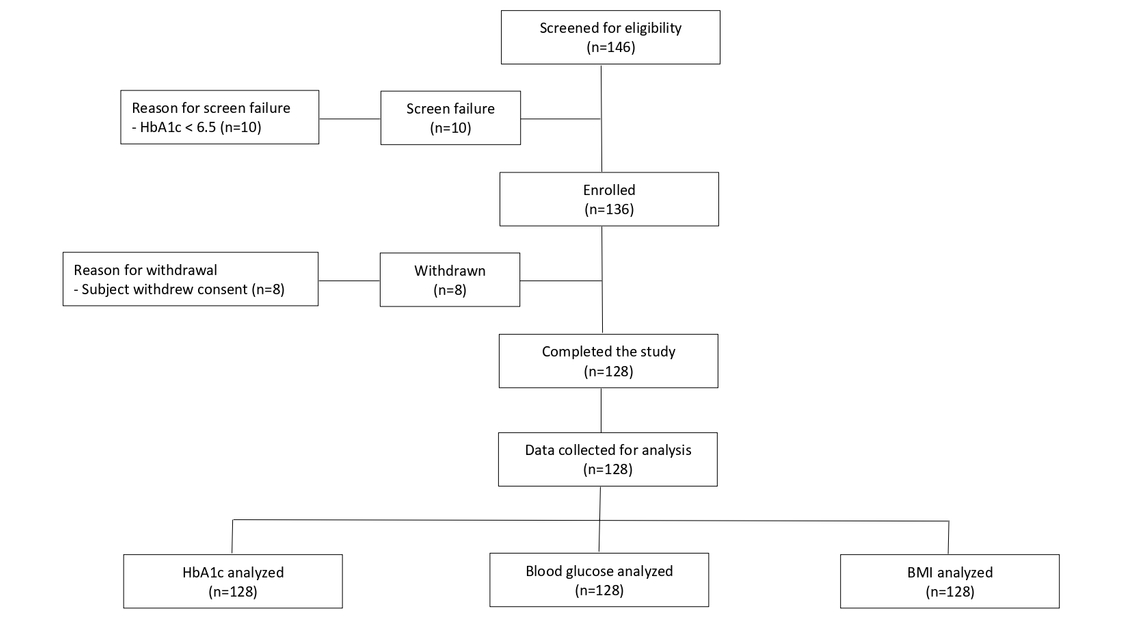
Participant recruitment and retention flowchart.

A total of 146 subjects were screened for eligibility and 136 meeting the eligibility criteria were included in the study (Figure 1). Of these 136 subjects, 8 withdrew consent during the study period for personal reasons. Final results were analyzed for the remaining 128 subjects who completed the intervention period of 12 weeks (Figure 1). The final study population had a mean age of 48.48 years, with a slight majority of males. Baseline characteristics of the participants are presented in Table 1.

**Table 1:** Baseline characteristics of the study subjects (N=128).

Characteristics		Value
Age (years), mean (SD)		48.48 (10.27)
**Age group (years), n (%)**		
	<30	3 (2.3)
	30-50	62 (48.4)
	>50	63 (49.2)
**Sex, n (%)**		
	Male	70 (54.7)
	Female	58 (45.3)
Weight (kg), mean (SD)		79.00 (16.12)
BMI (kg/m^2^), mean (SD)		29.13 (4.86)
**Blood pressure (mmHg), mean (SD)**		
	Systolic	125.07 (14.12)
	Diastolic	79.02 (8.20)
Heart rate (beats/min), mean (SD)		88.38 (10.73)
**Comorbid conditions, n (%)**		
	Hypertension	55 (42.9)
	COVID-19	33 (25.8)
	Thyroid disorder	10 (7.8)
	Respiratory disease	1 (0.8)
	Kidney disease	1 (0.8)
**Smoking history, n (%)**		
	Yes	15 (11.7)
	No	113 (88.3)
**Alcohol history, n (%)**		
	Yes	31 (24.2)
	No	97 (75.8)
**Dietary habit, n (%)**		
	Vegetarian	83 (64.8)
	Nonvegetarian	45 (35.2)
**Level of activity with respect to job/work, n (%)**		
	Sedentary	57 (44.5)
	Mildly active	53 (41.4)
	Moderately active	17 (13.3)
	Extremely active	1 (0.8)
**Stress level, n (%)**		
	Low	39 (30.5)
	Medium	54 (42.2)
	High	35 (27.3)

The general characteristics of T2DM of the participants at the time of entry into the study are presented in Table 2. Over 90% of the subjects had diabetes for ≥1 year. The mean baseline HbA_1c_ for participants was 8.32% and the mean baseline FBG was 139.16 mg/dl.

**Table 2:** Baseline type 2 diabetes mellitus (T2DM) characteristics of the study population (N=128).

Pattern of T2DM		Value
**Diabetes history, n (%)**		
	<1 year	12 (9.4)
	≥1 year	116 (90.6)
Family history of diabetes, n (%)		99 (77.3)
**Number of times diabetes medicine is missed in a month, n (%)**		
	0	106 (82.8)
	<4	15 (11.7)
	≥4	7 (5.5)
**Number of hypoglycemic events in a month, n (%)**		
	0	104 (81.3)
	<2	13 (10.2)
	≥2	11 (8.5)
**Frequency of blood sugar testing, n (%)**		
	<2 times a week	87 (67.9)
	≥2 times a week	41 (32.1)
HbA_1c_^a^ (%), mean (SD)		8.32 (1.48)
FBG^b^ (mg/dl), mean (SD)		139.16 (40.01)
PPBG^c^ (mg/dl), mean (SD)		175.30 (43.39)
HOMA-IR^d^ index, mean (SD)		5.39 (4.82)

^a^HbA_1c_: glycated hemoglobin.

^b^FBG: fasting blood glucose.

^c^PPBG: postprandial blood glucose.

^d^HOMA-IR: Homeostatic Model Assessment for Insulin Resistance.

### Outcome Measures

#### Change in HbA_1c_

At the end of the intervention period, 76.6% (98/128) of the subjects showed a reduced HbA_1c_ from the preintervention level (responders), whereas 23.4% (30/128) showed an HbA_1c_ level that was either the same or increased from the preintervention level (nonresponders). The mean decrease in HbA_1c_ for the complete study population was 0.84% (SD 1.36; *P*<.001) from a baseline value of 8.32% (SD 1.48) to 7.48% (SD 1.18) at the end of 12 weeks. The responder subgroup showed a reduction of 1.24% (SD 1.30; *P*<.001) in HbA_1c_ from 8.51% (SD 1.55) at baseline to 7.27% (SD 1.11) postintervention. Among the 98 responders, 33 (34%) had an HbA_1c_ reduction ≤0.5%, whereas 32 (33%), 11 (11%), and 22 (23%) showed a reduction of >0.5% to ≤1.0%, >1.0% to ≤1.5%, and >1.5%, respectively. Conversely, the nonresponders showed a significant increase in the mean HbA_1c_ level by 0.46% (SD 0.44; *P*<.001) from a mean baseline level of 7.70% (SD 1.05) to a postintervention level of 8.16% (SD 1.15).

#### Changes in FBG, PPBG, BMI, and HOMA-IR

Among the other outcome parameters, the mean FBG reduced by 8.39 mg/dl (SD 40.65; *P*=.02) from a baseline level of 139.16 mg/dl (SD 40.01), and the mean PPBG decreased by 14.97 mg/dl (SD 46.11; *P*<.001) from a baseline level of 175.30 mg/dl (SD 43.39). The mean BMI decreased by 0.24 kg/m^2^ (SD 1.40; *P*=.06), whereas the HOMA-IR index of the participants increased by 0.54 (SD 5.49; *P*=.29). Details are provided in Table A1 of Multimedia Appendix 1.

#### Impact of the Intervention on Self-Management of Diabetes

Among the 128 participants, 113 (88.3%) found a positive impact of the intervention program on their self-management of diabetes (*P*<.001), whereas 95.3% (122/128) of the participants found a positive impact of the reminders and nudges in improving their overall adherence to the diabetes treatment (*P*<.001).

#### Level of Program Engagement

We performed the engagement analysis only for the responders whose HbA_1c_ level was reduced at the end of the intervention (n=98). The summary of this analysis is shown in Table A2 of Multimedia Appendix 1. There was a reduction in mean HbA_1c_ by 1.31% (SD 1.45; *P*=.006) in the low-engagement group. In the medium-engagement group, there was a significant reduction in mean HbA_1c_, FBG, PPBG, and BMI by 1.16% (SD 1.22; *P*<.001), 15.49 mg/dl (SD 39.71; *P*=.02), 14.30 mg/dl (SD 32.20; *P*=.007), and 0.39 kg/m^2^ (SD 0.81; *P*=.003), respectively. Further, in the high-engagement group, there was also a significant reduction in mean HbA_1c_, FBG, and PPBG levels by 1.30% (SD 1.32; *P*<.001), 14.21 mg/dl (SD 43.69; *P*=.04), and 31.12 mg/dl (SD 56.45; *P*=.001), respectively.

#### Association of Individual Baseline Characteristics With HbA_1c_ Response

Logistic regression was performed to evaluate the association of baseline characteristics with achievement of HbA_1c_ response (Table 3). HbA_1c_ response showed a significant association with a baseline HbA_1c_ level of ≥7.5%, no prior history of smoking, no prior history of COVID-19 infection, as well as a medium and high level of program engagement.

**Figure 2:**
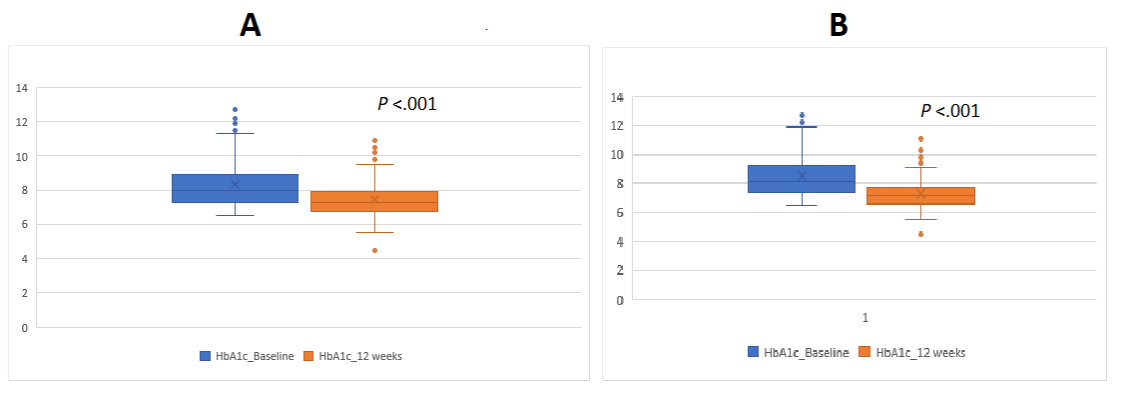
Comparison of HbA_1c_ levels. (A) Mean change in HbA_1c_ among all participants, (B) mean change in HbA_1c_ among those who had improvements in HbA_1c_ levels.

**Figure 3:**
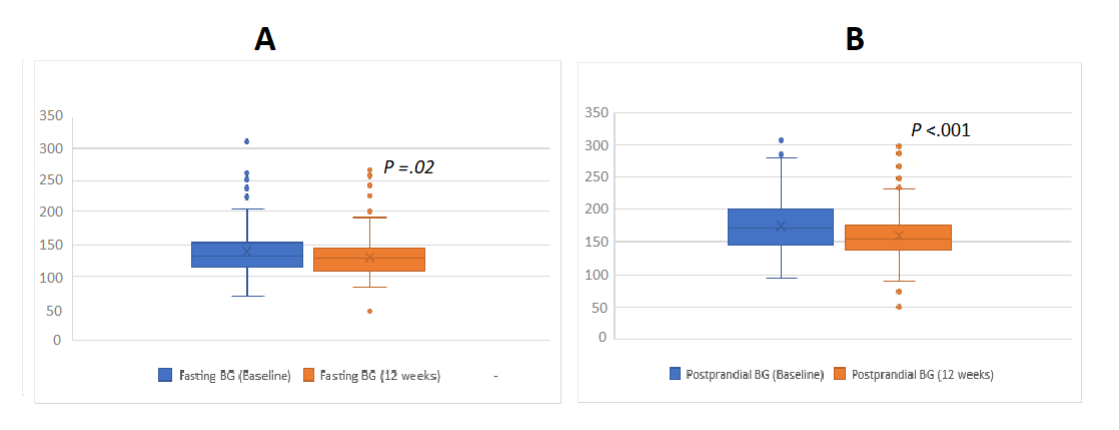
Change in blood sugar levels. Difference between (A) mean fasting blood glucose (BG) levels reported in the first week and final week of the intervention, and (B) mean postprandial BG levels reported in the first week and final week of the intervention.

Figure 2 presents the comparison of HbA_1c_ levels among different subgroups. The change in blood sugar levels among different subgroups is presented in Figure 3. Four participants had very low HbA_1c_, PPBG, and FBG values at 12 weeks. However, none of them had reported any episode of hypoglycemia during interactions with the care providers, and thus we suspect these instances to represent asymptomatic hypoglycemia episodes.

**Table 3:** Association of each baseline characteristic with achievement of glycated hemoglobin (HbA_1c_) response (N=128).

Variable			HbA_1c_ response				*χ*^2^ (*df*=21)			*P* value	
			Yes, n (%)		No, n (%)						
**Age (years)**								1.5			.22
	≤50	50 (51.0)		15 (50.0)							
	>50	48 (49.0)		15 (50.0)							
**Sex**								1.1			.28
	Male	54 (55.1)		16 (53.3)							
	Female	44 (44.9)		14 (46.7)							
**HbA_1c_ level (%)**								9.3			.002
	<7.5	25 (25.5)		16 (53.3)							
	≥7.5	73 (74.5)		14 (46.7)							
**BMI (** **kg/m** ^2^ **)**								0.3			.56
	<25	20 (20.4)		7 (23.3)							
	≥25	78 (79.6)		23 (76.7)							
**Smoking history**								4.8			.03
	Yes	10 (10.2)		5 (16.7)							
	No	88 (89.8)		25 (83.3)							
**Alcohol history**								1.3			.26
	Yes	24 (24.5)		7 (23.3)							
	No	74 (75.5)		23 (76.7)							
**Prior hypertension**								1.7			.19
	Yes	39 (39.8)		16 (53.3)							
	No	59 (60.2)		14 (46.7)							
**Prior COVID-19 infection**								11.7			<.001
	Yes	20 (20.4)		13 (43.3)							
	No	78 (79.6)		17 (56.7)							
**Dietary habits**								0.7			.41
	Vegetarian	60 (61.2)		23 (76.7)							
	Nonvegetarian	38 (38.8)		7 (23.3)							
**Family history of diabetes**								0.5			.49
	Yes	76 (77.5)		23 (76.7)							
	No	22 (22.5)		7 (23.3)							
**Diabetes history**								0.1			.83
	<1 year	12 (12.2)		0 (0.0)							
	≥1 year	86 (87.8)		30 (100.0)							
**Level of activity**								1.5			.83
	Sedentary	45 (45.9)		12 (40.0)							
	Mildly active	40 (40.8)		13 (43.3)							
	Moderately active	12 (12.2)		5 (16.7)							
	Extremely active	1 (1.0)		0 (0.0)							
**Stress level**								0.6			.74
	Low	29 (29.6)		10 (33.3)							
	Medium	43 (43.9)		11 (36.7)							
	High	26 (26.5)		9 (30.0)							
**Program engagement level**								7.3			.03
	Low	14 (14.2)		11 (36.7)							
	Medium	42 (42.9)		9 (30.0)							
	High	42 (42.9)		10 (33.3)							

## Discussion

### Principal Results and Comparison With Prior Work

This study has shown that patients with diabetes benefit from a component of lifestyle modification through digital means along with routine care with antidiabetic medication. Lifestyle modification through digital therapeutics resulted in a decrease in HbA_1c_, thereby bringing diabetes under control. In our study, a significant reduction in HbA_1c_ of 0.84% (SD 1.36; *P*<.001), in FBG of 8.39 mg/dl (SD 40.65; *P*=.02), and in PPBG of 14.97 mg/dl (SD 46.11; *P*<.001) were observed for the overall study population at the end of 12 weeks. These results are consistent with similar findings from multiple studies. A previous study from India that evaluated glycemic control in 102 patients with T2DM of South Asian origin using a digital therapeutic platform found a significant change (–0.49%, 95% CI −0.73 to 0.25; *P*<.001) in the mean HbA_1c_ level after 16 weeks of the intervention [12]. Berman et al [11] also showed a mean change in HbA_1c_ of –0.8% (SD 1.3; *P*<.001) after a 12-week intervention. A meta-analysis of 18 randomized controlled trials showed a significant change in mean HbA_1c_ levels (–0.54%, 95% CI –0.75 to –0.34; *P*<.05) with a telecare intervention [14]. A retrospective study of a digital health intervention in adults with T2DM also demonstrated a significant reduction in HbA_1c_ levels (–0.81%; *P*<.001) after 3 months [16].

A new approach is useful if the majority of patients are benefitting from it and if the benefit is highly probable. In our study, 76.6% (98/128) of the subjects benefitted from the digital intervention by achieving a reduction in their HbA_1c_ of 1.24% (SD 1.30; *P*<.001) from preintervention levels. This study used an inclusion criterion of HbA_1c_≥6.5% at baseline, thereby ensuring close to real-world representation of patients with diabetes in terms of HbA_1c_ levels, in contrast to similar effectiveness studies that have considered higher levels of baseline HbA_1c_ such as above 7.5% [15,19] or 8% [20] as one of the inclusion criteria. This may have reduced the effective postintervention mean change in HbA_1c_ for the total sample population. Yet, similar to studies by Wilson-Anumudu et al [15] and Krishnakumar et al [12], we also found that individuals with higher levels of baseline HbA_1c_ stand to benefit more from lifestyle intervention. This finding has significance by indicating that rather than escalating the medication dosage or type, such patients may be advised to undergo a lifestyle intervention to bring their HbA_1c_ levels under control or at manageable levels, without any significant side effects.

Comparison with other DTIs is difficult as these approaches differ with regard to the use of tools, frequency of interventions, and stakeholders involved, among other factors [21]. Hence, measurement of the impact of each DTI approach separately might be warranted. The DTI approach used in this study included a significant human component by way of involvement of the primary care physician of the patients, apart from dieticians and health coaches. The presence of a patient’s physician in the DTI care team may have created the psychological impact that the participant’s health is being monitored by their physician.

As seen in Table 3, there was a significant reduction in the mean BMI (–0.34 kg/m^2^; *P*=.007) in the responders subgroup. However, this reduction was relatively minimal compared with the observed change in HbA_1c_ levels (–1.24%; *P*<.001). Further subanalysis suggested the possibility of the impact of ongoing antidiabetic medications causing weight gain, as the majority (107/128) of the participants were on sulfonylureas, thiazolidinediones, alpha-glucosidase inhibitors, and insulin. Future studies may be needed to understand the confounding impacts of antidiabetic drugs causing weight gain and weight loss, and simultaneous lifestyle modification guided toward weight loss.

With regard to the program engagement level, we hypothesized that greater program engagement should result in greater improvement in postintervention blood glucose parameters. However, in this study, all three engagement groups showed statistically significant improvement in HbA_1c_ postintervention. In fact, the subjects in the medium-engagement group exhibited the smallest change in HbA_1c_ of –1.16% (SD 1.22), as compared to the low- and high-engagement groups with an HbA_1c_ reduction of –1.31% (SD 1.45) and –1.30% (SD 1.32), respectively. Although the linear reduction in HbA_1c_ in the medium- and high-engagement groups is in line with our hypothesis, the higher reduction in the low-engagement group could be attributed to the effect of a smaller sample size. This needs to be evaluated in future studies, as there is a difference between the mere arithmetic sum of engagement parameters vis-a-vis the compliance provided by the patient to engagement by the care team.

### Strengths and Limitations

The main limitations of our study are the lack of a control group, a short duration of follow-up to evaluate certain parameters such as BMI and HOMA-IR, and lack of measures to evaluate the compliance with respect to the level of program engagement. The prospective design of the study under a controlled environment, statistically derived sample size, and low dropout rate can be considered as the main strengths of the study. Further, one of the inclusion criteria of our study was patients on a stable dose of antidiabetic medication at the time of entry to the study, and they were expected to remain on the same stable dose during the study period. This minimized the scope of any potential observation bias and the baseline values of each subject served as their own control, thereby attributing the difference observed at the end of study to the study intervention. Further studies in this area in the form of randomized controlled trials are warranted.

### Conclusion

The use of a DTI as an adjunct therapy to conventional medications significantly reduced HbA_1c_, FBG, and PPBG levels in patients with T2DM. This in turn may reduce the risk of cardiovascular complications as well as all-cause mortality associated with T2DM.

## Appendix

Multimedia Appendix 1 Summary of outcome measures for responders and nonresponders (Table A1), and according to level of engagement (Table A2).

## Abbreviations

DTI: digital therapeutic intervention
FBG: fasting blood glucose
HbA_1c_: glycated hemoglobin
HOMA-IR: Homeostatic Model Assessment for Insulin Resistance
PPBG: postprandial blood glucose
SMBG: self-monitoring of blood glucose
T2DM: type 2 diabetes mellitus

## References

[1] IDF Diabetes Atlas, 10th edition. International Diabetes Federation.

[2] Patel SA, Shivashankar R, Ali MK, Anjana R, Deepa M, Kapoor D, Kondal D, Rautela G, Mohan V, Narayan KV, Kadir MM, Fatmi Z, Prabhakaran D, Tandon N, CARRS Investigators OBOT (2016). Is the "South Asian Phenotype" unique to South Asians?: Comparing cardiometabolic risk factors in the CARRS and NHANES studies. Glob Heart.

[3] Hsu W, Araneta M, Kanaya A, Chiang J, Fujimoto W (2015). BMI cut points to identify at-risk Asian Americans for type 2 diabetes screening. Diabetes Care.

[4] Unnikrishnan R, Anjana R, Mohan V (2014). Diabetes in South Asians: is the phenotype different?. Diabetes.

[5] Colberg SR, Sigal RJ, Fernhall B, Regensteiner JG, Blissmer BJ, Rubin RR, Chasan-Taber L, Albright AL, Braun B, American College of Sports Medicine, American Diabetes Association (2010). Exercise and type 2 diabetes: the American College of Sports Medicine and the American Diabetes Association: joint position statement. Diabetes Care.

[6] Ley SH, Hamdy O, Mohan V, Hu FB (2014). Prevention and management of type 2 diabetes: dietary components and nutritional strategies. Lancet.

[7] Cradock KA, ÓLaighin G, Finucane FM, McKay R, Quinlan LR, Martin Ginis KA, Gainforth HL (2017). Diet behavior change techniques in type 2 diabetes: a systematic review and meta-analysis. Diabetes Care.

[8] Katz DL, Frates EP, Bonnet JP, Gupta SK, Vartiainen E, Carmona RH (2018). Lifestyle as medicine: the case for a true health initiative. Am J Health Promot.

[9] Kim SH, Lee SJ, Kang ES, Kang S, Hur KY, Lee HJ, Ahn CW, Cha BS, Yoo JS, Lee HC (2006). Effects of lifestyle modification on metabolic parameters and carotid intima-media thickness in patients with type 2 diabetes mellitus. Metabolism.

[10] Adachi M, Yamaoka K, Watanabe M, Nishikawa M, Kobayashi I, Hida E, Tango T (2013). Effects of lifestyle education program for type 2 diabetes patients in clinics: a cluster randomized controlled trial. BMC Public Health.

[11] Berman MA, Guthrie NL, Edwards KL, Appelbaum KJ, Njike VY, Eisenberg DM, Katz DL (2018). Change in glycemic control with use of a digital therapeutic in adults with type 2 diabetes: cohort study. JMIR Diabetes.

[12] Krishnakumar A, Verma R, Chawla R, Sosale A, Saboo B, Joshi S, Shaikh M, Shah A, Kolwankar S, Mattoo V (2021). Evaluating glycemic control in patients of South Asian origin with type 2 diabetes using a digital therapeutic platform: analysis of real-world data. J Med Internet Res.

[13] Lemelin A, Godbout A, Paré G, Bernard S (2020). Improved glycemic control through the use of a telehomecare program in patients with diabetes treated with insulin. Diabetes Technol Ther.

[14] Huang Z, Tao H, Meng Q, Jing L (2015). Management of endocrine disease. Effects of telecare intervention on glycemic control in type 2 diabetes: a systematic review and meta-analysis of randomized controlled trials. Eur J Endocrinol.

[15] Wilson-Anumudu F, Quan R, Castro Sweet C, Cerrada C, Juusola J, Turken M, Bradner Jasik C (2021). Early insights from a digitally enhanced diabetes self-management education and support program: single-arm nonrandomized trial. JMIR Diabetes.

[16] Zimmermann G, Venkatesan A, Rawlings K, Scahill MD (2021). Improved glycemic control with a digital health intervention in adults with type 2 diabetes: retrospective study. JMIR Diabetes.

[17] Ku EJ, Park J, Jeon HJ, Oh T, Choi HJ (2020). Clinical efficacy and plausibility of a smartphone-based integrated online real-time diabetes care system via glucose and diet data management: a pilot study. Intern Med J.

[18] Bollyky JB, Bravata D, Yang J, Williamson M, Schneider J (2018). Remote lifestyle coaching plus a connected glucose meter with certified diabetes educator support improves glucose and weight loss for people with type 2 diabetes. J Diabetes Res.

[19] Quinn CC, Shardell MD, Terrin ML, Barr EA, Ballew SH, Gruber-Baldini AL (2011). Cluster-randomized trial of a mobile phone personalized behavioral intervention for blood glucose control. Diabetes Care.

[20] Agarwal P, Mukerji G, Desveaux L, Ivers NM, Bhattacharyya O, Hensel JM, Shaw J, Bouck Z, Jamieson T, Onabajo N, Cooper M, Marani H, Jeffs L, Bhatia RS (2019). Mobile app for improved self-management of type 2 diabetes: multicenter pragmatic randomized controlled trial. JMIR Mhealth Uhealth.

[21] Majithia AR, Kusiak CM, Armento Lee A, Colangelo FR, Romanelli RJ, Robertson S, Miller DP, Erani DM, Layne JE, Dixon RF, Zisser H (2020). Glycemic outcomes in adults with type 2 diabetes participating in a continuous glucose monitor-driven virtual diabetes clinic: prospective trial. J Med Internet Res.

