# Supplementary material for: Clinical Utility of a Digital Therapeutic Intervention in Indian Patients With Type 2 Diabetes Mellitus: 12-Week Prospective Single-Arm Intervention Study
Source: JMIR Diabetes. 2022 Oct 31;7(4):e41401. doi: 10.2196/41401 (PMC9664320; doi:10.2196/41401)
Supplement: Multimedia Appendix 1 [file diabetes_v7i4e41401_app1.docx]

**Table A1. Summary of Outcome Measures**

| **Outcome Measures** | **Total (N = 128)** | | | | **Responder (N = 98)** | | | | **Non-Responder (N = 30)** | | | |
| --- | --- | --- | --- | --- | --- | --- | --- | --- | --- | --- | --- | --- |
|  | **Pre-intervention** | **Post-intervention** | **Mean change** | ***P* value** | **Pre-intervention** | **Post-intervention** | **Mean change** | ***P* value** | **Pre-intervention** | **Post-intervention** | **Mean change** | ***P* value** |
| HbA1c, mean (SD), % | 8.32 (1.48) | 7.48 (1.18) | -0.84 (1.36) | **<.001** | 8.51 (SD 1.55) | 7.27 (SD 1.11) | -1.24 (SD 1.30) | **<.001** | 7.70 (SD 1.05) | 8.16 (SD 1.15) | 0.46 (SD 0.44) | **<.001** |
| FBG, mean (SD), mg/dl | 139.16 (SD 40.01) | 130.76 (SD 33.20) | -8.39 (SD 40.65) | **.02** | 139.54 (SD 42.08) | 127.12 (SD 30.84) | -12.42 (SD 40.47) | **.003** | 137.90 (SD 32.98) | 142.67 (SD 38.15) | 4.77 (SD 38.40) | .51 |
| PPBG, mean (SD), mg/dl | 175.30 (SD 43.39) | 160.33 (SD 39.47) | -14.97 (SD 46.11) | **<.001** | 176.21 (SD 45.98) | 154.76 (SD 37.46) | -21.46 (SD 45.11) | **<.001** | 172.30 (SD 34.09) | 178.53 (SD 40.99) | 6.23 (SD 42.85) | .44 |
| BMI, mean (SD), kg/m^2^ | 29.13 (SD 4.86) | 28.89 (SD 4.75) | -0.24 (SD 1.40) | .06 | 29.37 (SD 4.96) | 29.03 (SD 4.78) | -0.34 (SD 1.22) | **.007** | 28.34 (SD 4.49) | 28.44 (SD 4.70) | 0.11 (SD 1.83) | .76 |
| HOMA-IR Index, mean (SD), Ratio | 5.30 (SD 4.79) | 5.84 (SD 6.53) | 0.54 (SD 5.49) | .29 | 5.16 (SD 5.03) | 5.44 (SD 6.12) | 0.28 (SD 5.43) | .62 | 5.75 (SD 3.98) | 7.12 (SD 7.68) | 1.37 (SD 5.61) | .21 |

**Table A2. Summary of Outcome Measures among the Responders with the Level of Program Engagement (N = 98)**

| **Outcome Measures** | **Low Engagement Group (N = 14)** | | | | **Medium Engagement Group (N = 42)** | | | | **High Engagement Group (N = 42)** | | | |
| --- | --- | --- | --- | --- | --- | --- | --- | --- | --- | --- | --- | --- |
|  | **Pre-intervention** | **Post-intervention** | **Mean change** | ***P* value** | **Pre-intervention** | **Post-intervention** | **Mean change** | ***P* value** | **Pre-intervention** | **Post-intervention** | **Mean change** | ***P* value** |
| HbA1c, mean (SD), % | 8.59 (SD 1.53) | 7.29 (SD 0.89) | -1.31 (SD 1.45) | **.006** | 8.29 (SD 1.29) | 7.13 (SD 1.04) | -1.16 (SD 1.22) | **<.001** | 8.70 (SD 1.73) | 7.40 (SD 1.22) | -1.30 (SD 1.32) | **<.001** |
| FBG, mean (SD), mg/dl | 131.21 (SD 37.35) | 133.36 (SD 26.33) | 2.14 (SD 27.55) | .78 | 138.93 (SD 39.07) | 123.44 (SD 29.65) | -15.49 (SD 39.71) | **.02** | 142.93 (SD 45.41) | 128.71 (SD 32.51) | -14.21 (SD 43.69) | **.04** |
| PPBG, mean (SD), mg/dl | 168.43 (SD 39.98) | 154.50 (SD 19.82) | -13.93 (SD 32.96) | .15 | 169.40 (SD 39.94) | 155.10 (SD 33.09) | -14.30 (SD 32.20) | **.007** | 185.62 (SD 50.98) | 154.50 (SD 44.89) | -31.12 (SD 56.45) | **.001** |
| BMI, mean (SD), kg/m^2^ | 30.38 (SD 5.17) | 29.73 (SD 5.40) | -0.65 (SD 2.11) | .29 | 30.34 (SD 4.70) | 29.95 (SD 4.81) | -0.39 (SD 0.81) | **.003** | 28.06 (SD 4.77) | 27.87 (SD 4.20) | -0.19 (SD 1.13) | .29 |
| HOMA-IR Index, mean (SD), Ratio | 4.37 (SD 2.93) | 7.40 (SD 8.16) | 3.04 (SD 7.32) | .16 | 6.27 (SD 6.40) | 4.81 (SD 3.87) | -1.45 (SD 4.58) | .07 | 4.41 (SD 3.75) | 5.34 (SD 6.75) | 0.93 (SD 4.80) | .24 |
